# Exploratory study of macrophage polarization and spatial distribution in colorectal cancer liver metastasis: a pilot study

**DOI:** 10.3389/fimmu.2023.1223864

**Published:** 2023-08-10

**Authors:** Isha Khanduri, Dipen M. Maru, Edwin R. Parra

**Affiliations:** ^1^ Department of Translational Molecular Pathology, The University of Texas MD Anderson Cancer Center, Houston, TX, United States; ^2^ Department of Pathology, The University of Texas MD Anderson Cancer Center, Houston, TX, United States

**Keywords:** macrophages, multiplex immunofluorescence, spatial, colorectal cancer, liver macrophages, liver

## Abstract

**Background:**

The liver is the most typical site of metastatic disease for patients with colorectal cancer (CRC), and up to half the patients with CRC will develop colorectal liver metastasis (CLM). Studying the tumor microenvironment, particularly macrophages and their spatial distribution, can give us critical insight into treatment.

**Methods:**

Ten CLMs (five treatment-naïve and five post–neoadjuvant chemotherapy) were stained with multiplex immunofluorescence panels against cytokeratins, CD68, Arg1, CD206, CD86, CD163, PD-L1, and MRP8-14. Densities of cell phenotypes and their spatial distribution in the tumor center and the normal liver–tumor interface were correlated with clinicopathological variables.

**Results:**

M2 macrophages were the predominant subtype in both the tumor center and the periphery, with a relatively higher density at the periphery. The larger tumors, more than 3.9 cm, were associated with higher densities of total CD68+ macrophages and CD68+CD163+ CD206^neg^ and CD68+CD206+ CD163^neg^ M2 macrophage subtypes. Total macrophages in the tumor periphery demonstrated significantly greater proximity to malignant cells than did those in the tumor center (p=0.0371). The presence of higher than median CD68+MRP8-14+CD86^neg^ M1 macrophages in the tumor center was associated with poor overall survival (median 2.34 years) compared to cases with lower than median M1 macrophages at the tumor center (median 6.41 years) in univariate analysis.

**Conclusion:**

The dominant polarization of the M2 macrophage subtype could drive new therapeutic approaches in CLM patients.

## Introduction

Colorectal cancer (CRC) is the third most common malignancy and the second leading cause of cancer deaths worldwide (1). Moreover, 35%-55% of CRC patients develop hepatic metastases during their disease (2). Cytotoxic chemotherapy, in combination with biologically targeted agents, is the first-line therapy recommended for metastatic colorectal cancer (3). Targeted therapy offers several advantages like less toxicity and higher efficacy compared with conventional chemotherapy and hence should be further explored as a treatment modality (4, 5). The study of the colorectal liver metastasis (CLM) tumor microenvironment (TME) is pivotal to discover novel therapeutic biomarkers for treatment of CLM (6, 7).

Immunohistochemistry is an efficient and routine tool for pathological analysis of important clinical markers. Standard immunohistochemistry typically allows the analysis of a single marker per slide, but this limitation has been overcome with the recent advent of multiplexed imaging technologies, such as multiplex immunofluorescence (mIF) tyramide signal amplification. These tools can reliably decipher the TME via simultaneous detection of multiple markers on tumor and immune cells in a single tissue section (8–11). The significance of these techniques is augmented by limited tissue availability for research purposes and an increasing demand for extensive analysis (12).

In addition to detection of cell phenotypes, mIF facilitates analysis of spatial distribution of tumor cells and tumor-associated immune cells. Data extracted from mIF-based digital image analysis allows for detailed characterization of cell-cell associations and the geographic distribution of cell phenotypes, which may help predict clinical responses and mechanisms of cancer resistance to immunotherapies (13).

This study aims to analyze in depth the TME of CLM in the context of tumor-associated macrophages (TAMs). Using mIF, we sought to identify the polarization of TAMs into subtypes and their spatial distribution in the TME and to determine these subtypes’ prognostic significance.

## Materials and methods

### Tissue specimen

The study included 10 randomly selected colorectal carcinoma patients with liver metastasis who underwent liver resection surgery. Patients’ ages, demographics, clinical and pathological characteristics, preoperative chemotherapy types, and degree of pathologic response to chemotherapy were retrieved from a review of electronic medical records (**Table 1**) for correlation with mIF data. In addition, 4-µm-thick sections of formalin-fixed, paraffin-embedded (FFPE) CLM tissue from resection specimens were prepared for analysis by the mIF platform.

**Table 1:** Clinicopathological and demographic characteristics of the patient population (n=10).

Characteristic	Value
**Age, median (range), years**	60 (38-79.5)
Sex	
Male	6
Female	4
Pre-surgery treatment	
FOLFOX + bevacizumab	5
Treatment naïve	5
Positive lymph node, primary tumor	
Yes	7
No	3
CLM synchronous to primary	
Yes	3
No	7
**Preoperative serum CEA**, median (range), ng/ml	36.05 (0.5-438.2)
**Diameter of largest CLM**, median (range), cm	3.9 (1.7-14)
**Recurrence-free survival**, median (range), months	11.8 (4.3-143.8)
**Overall survival**, median (range), months	60 (11.5-143.8)

**Figure 1:**
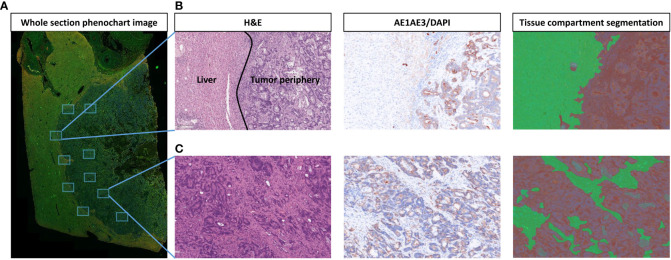
Strategy for selection and segmentation of regions of interest (ROIs). **(A)** Whole section image of liver tissue with colorectal liver metastasis (CLM) in Phenochart 1.0.12 image viewer software with 5 ROIs selected each from the tumor center and tumor–normal liver interface (periphery). **(B)** Representative image from tumor periphery with hematoxylin and eosin (H&E), single-stained cytokeratin (CK), and tissue compartment segmentation (green: normal liver, red: tumor periphery). **(C)** Representative image from tumor center with H&E, single-stained CK, and tissue compartment segmentation (green: stroma, red: tumor). Images were generated using Vectra Polaris 1.0.13 scanner system and InForm 2.4.8 image analysis software (Akoya Biosciences).

Two pathologists reviewed hematoxylin and eosin–stained sections to select blocks with the highest tumor cellularity and intact tumor-normal interface for mIF analysis. Regions of interest (ROIs) from representative areas of the tumor center were selected, and regions on the tumor periphery were selected to represent the interface between the CLM and the adjacent normal liver tissue (**Figure 1**). Foci of necrosis and extensive post-therapy changes were excluded from the analysis.

### mIF staining and analysis

Automated mIF staining was performed on 4-µm-thick FFPE representative tumor sections using previously described and validated protocols (14–16). The immunofluorescence markers were grouped into one 8-antibody panel for characterization of macrophage populations (**Supplementary Table 1**).

**Figure 2:**
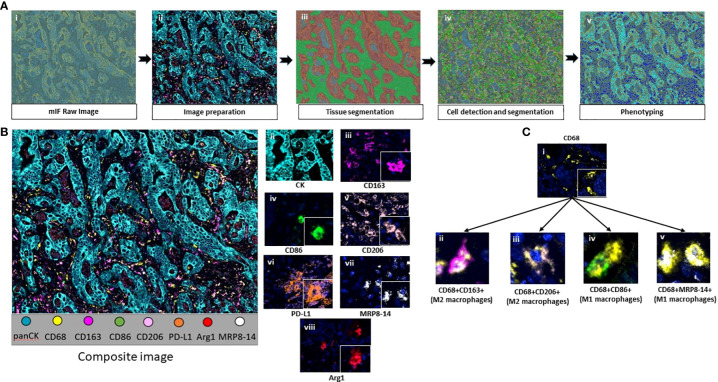
**(A)** Workflow of mIF digital image analysis. After image scanning, raw images (i) were prepared by activating the fluorochromes attached to the surface proteins (ii). Tissue segmentation was performed by training the software using representative examples from each compartment (iii). Cell limits were defined, and cells were individually identified (iv). Phenotyping of cells based on expression of surface proteins (v). **(B)** Composite image of tumor center after image preparation (i) and representative examples of all the markers included in the macrophage panel (ii-viii). **(C)** CD68+ macrophages (i) and their colocalization examples (ii-v). Images were generated using Vectra Polaris 1.0.13 scanner system and InForm 2.4.8 image analysis software (Akoya Biosciences).

The CLM samples were initially scanned at low magnification (10×) using Vectra Polaris 1.0.13, a multispectral imaging system (Akoya Biosciences, Marlborough, MA). Phenochart 1.0.12 image viewer software was used by a pathologist to select five 660×500 μm ROIs from the tumor center and five more from the tumor-normal interface (periphery) of each sample to capture representative areas with tissue heterogeneity. The areas identified for analysis were then scanned at a higher magnification of 200× (931×698 µm at resolution 20×, 0.5 µm/pixel) for further analysis. inForm 2.4.8 image analysis software (Akoya Biosciences) was then used to analyze each ROI by detailed tissue and cell segmentation and co-localization of antibodies in the mIF panel (15). Each ROI from the tumor center was segmented into two compartments: tumor, composed of the solid and glandular epithelial component, and stroma, composed of the fibrous connective tissue amidst the epithelial component. Similarly, ROIs from the periphery were segmented into the tumor compartment, composed of the peripheral tumor region, and the normal liver tissue adjacent to the tumor (**Figure 1**). Various macrophage phenotypes were identified by co-expression of the markers in the panel (**Supplementary Table 2**). **Figure 2** shows the workflow of digital image analysis by mIF.

### CLM spatial distribution analysis

To explore the geographical distribution and positioning of the macrophage phenotypes relative to the tumor cells, spatial analysis of the cell phenotypes was performed using RStudio 3.5.3 (phenoptr 0.2.2 packet, Akoya Biosciences). Analysis of each image yields x and y coordinates for each cell phenotype. These coordinates were used to calculate the distance between each malignant cell and each macrophage phenotype and assist in mapping the distribution pattern of each phenotype in the tissue. We applied the median nearest neighbor function from malignant cells to different macrophage phenotypes to determine whether the macrophages were close to (i.e., equal to or less than the median distance) or far from (i.e., more than the median distance) the malignant cells. This information was then compared with clinicopathological features to determine the potential role of spatial distribution of cells in treatment response and patient prognosis (17, 18).

### Statistical analysis

To evaluate if the densities of biomarkers or cellular distribution was prognostically associated with survival, we dichotomized biomarker densities and distances by the median and used the log-rank test to perform univariate survival analysis using recurrence-free survival and overall survival. Recurrence-free survival was defined as the interval from surgery to recurrence or last contact, and overall survival was defined as the interval from surgery to death or last contact. To assess whether continuous biomarker data were associated with clinical variables, we used nonparametric tests: Spearman’s rank correlation for continuous clinical variables, Mann-Whitney U test for categorical clinical variables with two groups, and Kruskal-Wallis test for categorical clinical variables with more than two groups. P-value of less than 0.05 was considered statistically significant.

## Results

### Immunophenotyping characteristics of macrophages in CLM

Using the mIF panel, we performed a quantitative analysis of the TAMs in CLM. **Table 2** shows the distribution of total macrophages and their subtypes in the tumor center and periphery. Among the macrophage subtypes, CD68+CD163+ CD206^neg^ and CD68+CD206+ CD163^neg^ M2 macrophages had higher densities than the CD68+CD86+ MRP8-14^neg^ and CD68+MRP8-14+CD86^neg^ M1 macrophages, in both the tumor center and periphery. Overall, there was a lack of M1 macrophages in the CLM TME. In addition, minimal expression of Arg1 and MRP8-14 on macrophages hindered the ability to further subclassify the M2 macrophages into the M2a, M2b, and M2c subtypes. Similarly, PD-L1 expression on macrophages was noted in only one of the 10 cases, precluding further analysis and correlation of PD-L1 expression on macrophages with clinicopathological features.

**Table 2:** Macrophage population density in the tumor center compared with adjacent normal liver and tumor periphery.

Macrophage phenotype, median (range), number per mm^2^	Tumor center	Peripheral normal liver	p for tumor center vs. liver	Tumor center	Tumor periphery	p for tumor center vs. periphery (tumor)
**Total macrophages (CD68+)**	12.96 (3.55-62.29)	12.69 (2.39-93.26)	0.739	12.96 (3.55-62.29)	68.28 (6.83-193.09)	**0.019**
**M1 (CD68+ MRP8-14+ CD163^neg^ CD206 ^neg^ Arg-1 ^neg^)**	0 (0-0.78)	0 (0-21.54)	0.353	0 (0-0.78)	0 (0-19.97)	0.721
**M1 (CD68+ CD86+CD163^neg^ CD206^neg^Arg1 ^neg^)**	0 (0-0.34)	0 (0-0.47)	0.739	0 (0-0.34)	0 (0-1.23)	0.3125
**M1 (CD68+ CD86+ MRP8-14+ CD163^neg^CD206 ^neg^ Arg1 ^neg^)**	0 (0)	0 (0)	–	0 (0)	0 (0)	–
**M2 (CD68+ CD163+ MRP8-14 ^neg^CD86 ^neg^)**	1.33 (0-16.93)	3.43 (0-54.34)	0.165	1.33 (0-16.93)	2.49 (0-77.05)	0.388
**M2 (CD68+ CD206+ MRP8-14 ^neg^ CD86 ^neg^)**	0.15 (0-1.96)	0.23 (0-22.3)	0.631	0.15 (0-1.96)	0.29 (0-38.05)	0.5781
**M2 (CD68+ CD163+ CD206+ MRP8-1^neg^ CD86 ^neg^)**	0 (0-0.78)	0 (0-21.05)	0.280	0 (0-0.78)	0 (0-19.97)	0.500
**CD68+ PD-L1+**	0 (0-0.37)	0 (0-0.47)	1.000	0 (0-0.37)	0 (0-2.46)	0.99

Note: Boldface indicates statistically significant differences.

### Macrophage subtype densities in the tumor center and periphery

Total macrophages and their subtypes were compared between the tumor center and the peripheral region, comprising both the tumor tissue in the peripheral region and the normal liver immediately adjacent to the tumor (**Table 2**).

**Figure 3:**
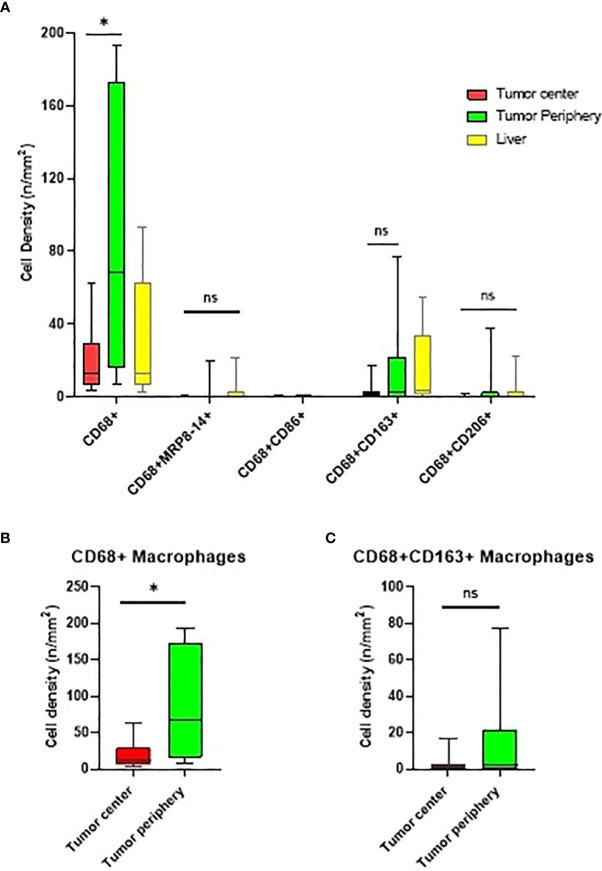
Box and whisker plots representing the distribution of tumor-associated macrophage (TAM) population densities across the different regions of interest. **(A)** Distribution of TAMs in the tumor center, tumor periphery, and normal liver. **(B, C)** Comparison of CD68+ and CD68+CD163+CD206^neg^ macrophage densities in the tumor center and tumor periphery. * significant P value; ns, no-significant. Graphs were generated using GraphPad Prism software version 9.0.0.

The tumor periphery demonstrated significantly higher densities of total CD68+ macrophages (median of 68.28 cells/mm^2^) compared to the tumor center (12.96 cells/mm^2^) (**Figure 3**). On the other hand, there was no significant difference in the distribution of CD68+CD163+ CD206^neg^ M2, CD68+CD86+MRP8-14^neg^ M1 or CD68+MRP8-14+ CD86^neg^ M1 macrophages between the tumor periphery and tumor center.

Upon comparison of the tumor center to the adjacent liver tissue, we found no significant differences in the densities of macrophage subtypes.

Interestingly, tumors larger than the median size (3.9 cm) demonstrated significantly higher densities of total macrophages, as well as CD68+CD163+ CD206^neg^ and CD68+CD206+CD163^neg^ M2 macrophage subtypes, in the tumor periphery and significantly higher densities of total, CD68+MRP8-14+ CD86^neg^ M1, and CD68+CD163+ CD206^neg^ and CD68+CD206+ CD163^neg^ M2 macrophages in the adjacent liver compared to smaller tumors (**Table 3**).

**Table 3:** Macrophage population densities according to tumor size in the tumor periphery and adjacent normal liver.

Macrophage phenotype median (range) number per mm^2^	Tumor periphery		p	Peripheral region, liver		p
	Tumor size <3.9 cm	Tumor size ≥3.9 cm		Tumor size <3.9 cm	Tumor size ≥3.9 cm	
**Total macrophages (CD68+)**	16.86 (0-68.03)	168.69 (68.53-193.09)	**0.008**	7.89 (2.39-13.11)	55.84 (9.82-93.26)	**0.032**
**M1 (CD68+ MRP8-14+ CD163^neg^CD206^neg^ Arg1 ^neg^)**	0 (0)	0 (0-19.97)	0.444	0 (0)	0.95 (0-21.54)	**0.032**
**M1 (CD68+CD86+ CD163^neg^CD206^neg^ Arg1 ^neg^)**	0 (0-1.02)	0 (0-1.23)	0.841	0 (0)	0 (0-0.47)	0.690
**M1 (CD68+CD86+ MRP8-14+CD163^neg^ CD206 ^neg^ Arg1 ^neg^)**	0 (0)	0 (0)	–	0 (0)	0 (0)	–
**M2 (CD68+CD163+ MRP8-14 ^neg^ CD86 ^neg^)**	0 (0-3.05)	20.37 (1.94-77.05)	**0.016**	1.32 (0-5.46)	31.31 (1.40-54.34)	**0.016**
**M2 (CD68+CD206+ MRP8-14 ^neg^ CD86 ^neg^)**	0 (0-0.58)	1.85 (0-38.05)	**0.048**	0 (0-0.45)	0.54 (0-22.03)	0.056
**M2 (CD68+CD163+ CD206+MRP8-14^neg^ CD86 ^neg^)**	0 (0)	0 (0-19.97)	0.444	0 (0)	0.54 (0-21.05)	**0.032**
**CD68+ PD-L1+**	0 (0)	0 (0-2.46)		0 (0)	0 (0-21.05)	0.690

Note: Boldface indicates statistically significant differences.

### Spatial distribution of macrophages

Data obtained using the mIF platform were used to explore the spatial distribution of the macrophage phenotypes. The spatial orientation of macrophages and their subtypes was based on their distance from cytokeratin-positive (CK+) malignant cells (MCs). The total macrophage population in the tumor center was a median of 159.07 μm from MCs, and the total macrophage population in the tumor periphery was a median of 83.69 μm from MCs. These distances were used to categorize macrophage subtypes as near (within the median distance) and far (outside the median distance) from the MCs (15).

**Figure 4:**
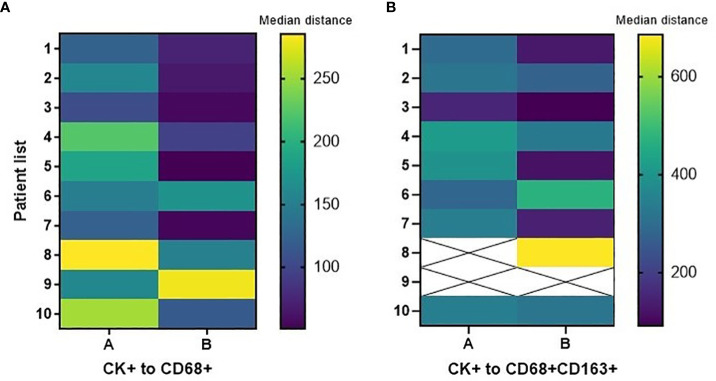
Heatmaps displaying median distances of cytokeratin-positive (CK+) malignant cells to total CD68+ macrophages and CD68+CD163+CD206^neg^ M2 macrophages in the tumor center **(A)** and tumor periphery **(B)** in 10 CLM patients. The images were generated using GraphPad Prism software version 9.0.0.

In the tumor center and in the tumor periphery, the CD68+CD86+MRP8-14^neg^ M1, CD68+CD163+ CD206^neg^ M2, and CD68+CD206+CD163^neg^ M2 macrophage subtypes were far from the MCs (**Table 4**). The total macrophages in the tumor periphery were significantly closer to MCs in the tumor periphery compared to those in the tumor center (p=0.0371). We observed a similar trend with the CD68+CD163+CD206^neg^ M2 macrophages; however, this did not reach significance. **Figure 4** shows the median distances of MCs to total and CD68+CD163+CD206^neg^ M2 macrophages.

**Table 4:** Median distances from malignant cells (CK+) to the macrophage phenotypes in the tumor center and tumor periphery.

Macrophage phenotype	Distance from CK+ cells, μm	
	Tumor center	Tumor periphery
Total macrophages (CD68+)	159.07	83.69
M1(CD68+CD86+CD163**^neg^** CD206**^neg^** Arg1**^neg^**)	325.3	320.5
M2 (CD68+CD163+MRP8-14 **^neg^** CD86 **^neg^**)	330.6	276.9
M2 (CD68+CD206+MRP8-14 **^neg^** CD86 **^neg^**)	456.1	369.6

Note: Macrophage populations in the tumor center within 159.07 μm of malignant cells and macrophage populations in the tumor periphery within 83.69 μm of malignant cells were considered near the malignant cells.

**Figure 5:**
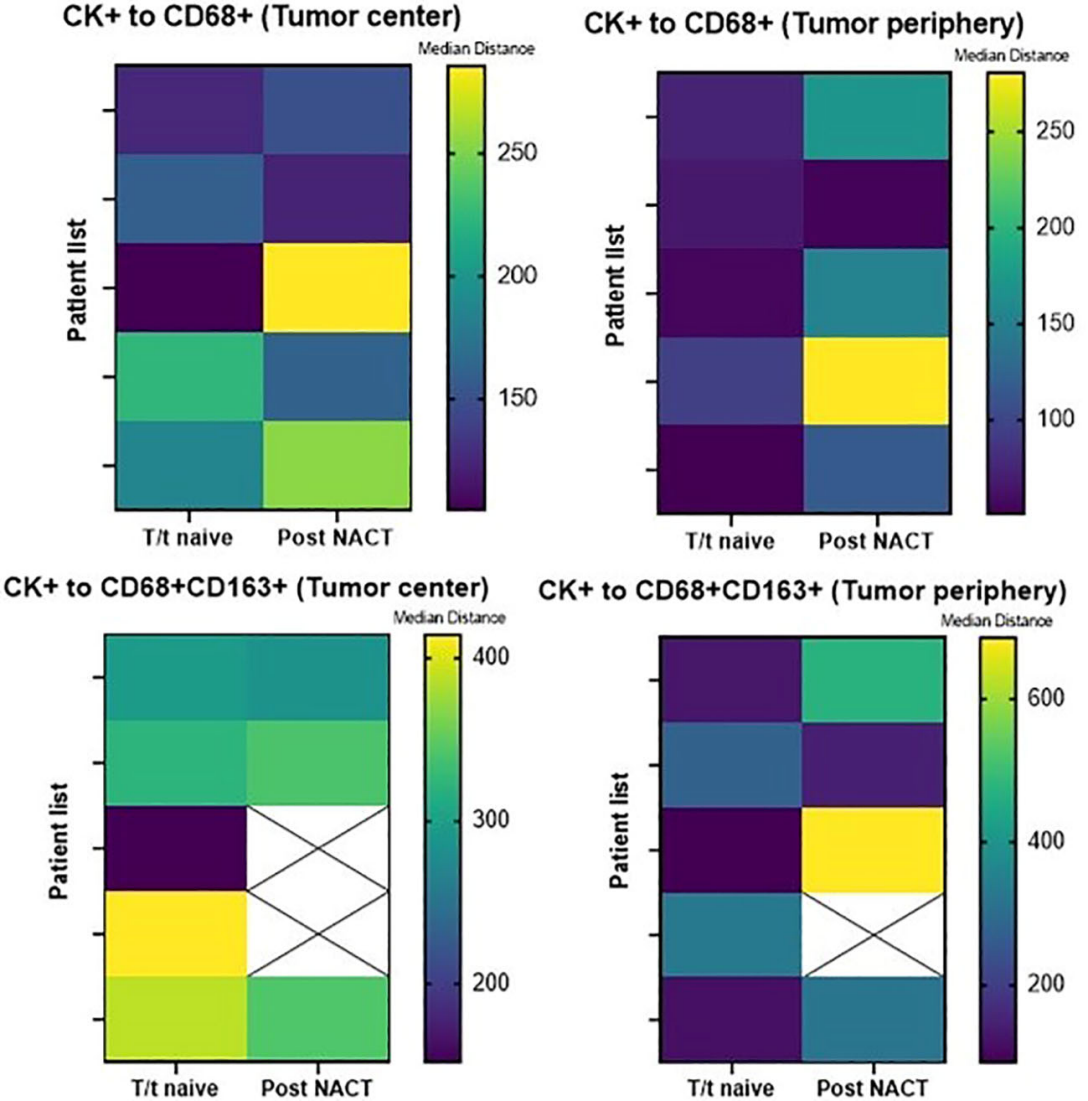
Heatmaps displaying median distances of CK+ malignant cells to total CD68+ macrophages and CD68+CD163+CD206^neg^ M2 macrophages in the tumor center and tumor periphery in 5 treatment-naïve patients and 5 post–neoadjuvant chemotherapy (NACT) patients. The images were generated using GraphPad Prism software version 9.0.0. T/t, treatment.

There was no significant difference in the median distance of CK+ MCs to total macrophages and M2 macrophages between treatment-naïve patients and patients treated with FOLFOX + bevacizumab neoadjuvant chemotherapy, in the tumor center and in the tumor periphery. **Figure 5** shows median distances of MCs to total and CD68+CD163+ CD206^neg^ M2 macrophages in these treatment groups by tissue region.

### Survival analysis

**Figure 6:**
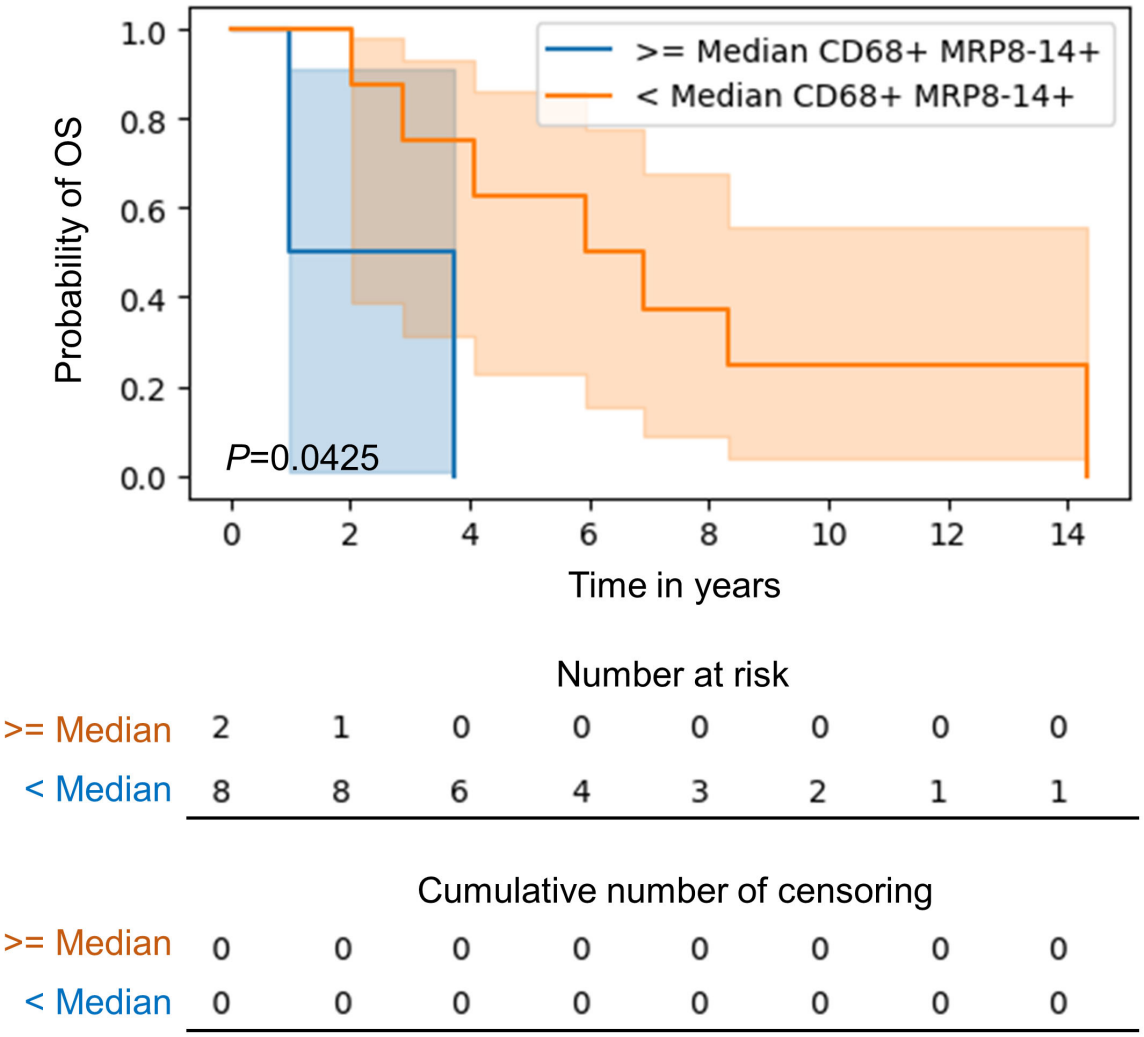
Kaplan-Meier curve of overall survival for patients with higher than median as compared to lower than median CD68+MRP8-14+ M1 macrophages in the tumor center in CLM. Kaplan‐Meier curves and log-rank test were generated by the R studio software version 3.6.0.

In our comparison of macrophage densities and patient survival, higher densities (median density used as a cuff off) of CD68+MRP8-14+CD86^neg^ M1 macrophages in the tumor center was associated with a poorer overall survival (median 2.34 years) compared to the lower densities of this subtype (median 6.41 years) in univariate analysis (**Figure 6**). There was no further association of total macrophage or macrophage subtype density or spatial distribution with patient overall or recurrence free survival. Survival did not significantly differ between the treatment-naïve and post–neoadjuvant chemotherapy patients.

## Discussion

In this exploratory study we identified that CD68+CD163+CD206^neg^ M2 macrophages were the predominant macrophage subtype in CLM, predominantly located in the tumor periphery with relatively higher density than the tumor center supported by the cellular spatial analysis. On the other hand, we observed that higher densities of CD68+MRP8-14+CD86^neg^ M1 macrophages in the tumor center is associated with poor overall survival compared with the low densities in CLM. The TME of CLM in the context of the macrophage population is still an area under exploration. Using techniques like mIF can help quantitate the macrophage subtypes and determine their spatial relationships, offering novel insights into the heterogeneity and complex functions of TAMs. In addition, using this data to determine the relative geographical distribution of cancer and immune cells helps us explore their biological interactions.

We used an mIF panel specifically curated for identifying macrophages and their subtypes to study macrophages’ polarization and spatial distribution in resected specimens from 10 CLM cases with and without neoadjuvant chemotherapy. The total macrophage density was highest in the tumor periphery compared to the tumor center and the adjacent normal liver. Yoshikawa et al. (19) and Huang et al. (20) also found higher median counts of CD68+ macrophages in the pancreatic ductal carcinoma and gastric adenocarcinoma periphery respectively compared to the center. Although it is well established in the literature that tumor margin demonstrates higher TAM densities, their role at the invasive tumor front is still controversial. On the one hand, TAMs facilitate invasive tumor potential by expression of Cathepsin B and S and matrix-degrading enzyme MMP-9 in gastric and pancreatic tumors, and on the other hand, in colon carcinoma TAMs at the invasive front have an anti-tumor role via expression of CD80 and CD86 (T cell activating costimulatory signals) (21). It is hypothesized that TAMs at the invasive front in CLM exhibit an immunosuppressive role by PD-L1 expression (22).The tumor periphery also had relatively higher density of CD68+CD163+CD206^neg^ M2 macrophages than the center. However, interestingly, the highest median density of CD68+CD163+CD206^neg^ M2 macrophages was seen in the normal adjacent liver. Studies (23, 24) suggest that activated Kupffer cells (liver tissue–resident macrophages) may co-express M2 macrophage markers CD68 and CD163, which may explained this increase of M2 macrophages in the adjacent liver.

TAMs exhibit a dual role in the TME. IFN-Y induces polarization of TAMs, TNF-α, and lipopolysaccharide (LPS) into the M1 (classically activated/anti-tumor) macrophage phenotype and IL-4, IL-190, TGFβ1, and PGE2, into the M2 (alternatively activated/pro-tumor) macrophage phenotype (25–28). In our analysis, M2 macrophages were the predominant subtype in the CLM TME compared to the M1 subtype in all tumor regions analyzed, suggesting an immunosuppressive environment in CLM. The fact that TAMs predominantly exhibit an M2 phenotype has been extensively reported in the literature (29–31). Although M2 was the dominant subtype, we found only a small fraction of total macrophages expressing M2 markers. Furthermore, we could not subtype the M2 macrophages into M2a, M2b, and M2c owing to a lack of Arg1 and MRP8-14 expression.

Interestingly, in the peripheral region, there was a remarkable increase in the density of total, CD68+MRP8-14+CD86^neg^ M1, CD68+CD163+CD206^neg^ M2, and CD68+CD206+CD163^neg^ M2 macrophages among tumors ≥3.9 cm (that is, larger than median) compared to tumors <3.9 cm. However, this comparison did not yield significant results when made in the tumor center. Following our observation of higher TAM density at the tumor periphery compared to the tumor center, this finding supports the hypothesis that TAMs have an immunosuppressive role at the advancing tumor front facilitating tumor invasion. In addition, studies have reported the correlation of macrophage densities with larger tumor size in primary breast cancer (32–34) and pancreatic adenocarcinoma (19).

While CD68+MRP8-14+CD86^neg^ M1 macrophages correlated with poorer overall survival in our cohort, this was not confirmed in a multivariate model. This is an exciting and novel finding as, M1 macrophages have been established as an anti-tumor macrophage subtype, however, their precise role in tumor progression can vary depending on various factors, including the tumor microenvironment, tumor type, and stage of tumor development. The role of M1 macrophages in tumor progression has recently been studied by Podlesnaya et al., who hypothesize that cytotoxic M1 macrophage activity may promote tumor progression via facilitating tumor immune escape (35) and in esophageal squamous cell carcinoma via GDF15-mediated ErbB2 phosphorylation (36). Additionally, increasing evidence supports the correlation of M2 macrophages with better prognosis. It elicits a partial tumor-limiting effect by facilitating vascular maturation and inhibiting intravasation of tumor cells into the vasculature (20, 37). It is important to note that the role of macrophages in tumor is complex and context dependent. The balance between M1 and M2 macrophages within the tumor microenvironment can determine the outcome of tumors growth and metastasis. A small cohort is one of the limitations of our study, and a larger cohort is required to confirm our results. No correlation was observed between patient survival and the proximity of macrophages to MCs.

We applied spatial analysis in an exploratory manner to map the spatial relationships between macrophages and MCs. The total macrophages in the peripheral region were significantly closer to MCs compared to the tumor center, suggesting that macrophages have a more activating or inhibitory influence over MCs in the tumor periphery. CD68+CD163+CD206^neg^ M2 macrophages demonstrated the same trend, although it was not significant. These two observations are in concordance with studies showing that TAMs have pro-tumor effects and are associated with tumor progression by aiding angiogenesis (38, 39). The proximity of macrophages to MCs may also potentiate epithelial-mesenchymal transition of tumor cells in the tumor periphery (40), leading to tumor progression. We did not find any correlation between the proximity of the macrophage subtypes to MCs and any other clinicopathologic features.

As previously mentioned, the small number of cases was a significant limitation in our study. A larger cohort is needed to validate our results and to better understand the TAM population. Additionally, the inability to classify a substantial fraction of macrophages as either of the subtypes left us with the question of whether that population was composed of non-polarized (M0) macrophages or expressed markers not included in our limited panel. Other techniques (41, 42) might help classify this population using various markers. The shallow expression of MRP8-14, Arg1, and PD-L1 on macrophages also hindered subclassifying the M2 subtype and studying the significance of PD-L1 expression on macrophages in CLM. Some studies have demonstrated high PD-L1 expression on macrophages in gastric cancer (32) and non–small cell lung cancer (43).

## Conclusions

In summary, in our exploratory study comprehensively analyzing the role of TAMs in CLM patients, TAMs contributed to an immunosuppressive environment. We also observed the heterogeneity of the macrophage population in different tumor regions, with a more robust macrophage response seen at the tumor periphery, which was supported by spatial analysis. However, the role of TAMs in CLM is complex, and the fact that macrophages display plasticity indicates that they might not be confined to the M1/M2 model. We emphasize using multiplex techniques to further investigate the role of macrophages in tumor progression and resistance to therapies and to potentially determine novel therapeutic candidates.

## Data availability statement

The original contributions presented in the study are included in the article/**Supplementary Material**. Further inquiries can be directed to the corresponding author.

## Ethics statement

The studies involving humans were approved by MD Anderson Institutional Review Board. The studies were conducted in accordance with the local legislation and institutional requirements. The participants provided their written informed consent to participate in this study.

## Author contributions

ERP and DMM contributed to the study conception. IK and ERP were responsible for writing the manuscript. ERP and IK conducted the data analysis. All authors contributed to the article and approved the submitted version.
