# Supplementary material for: Exploratory study of macrophage polarization and spatial distribution in colorectal cancer liver metastasis: a pilot study
Source: Front Immunol. 2023 Aug 10;14:1223864. doi: 10.3389/fimmu.2023.1223864 (PMC10449458; doi:10.3389/fimmu.2023.1223864)
Supplement: Supplementary file 1 [file Table_1.docx]

**Supplementary Material**

Exploratory Study of Macrophage Polarization and Spatial Distribution in Colorectal Cancer Liver Metastasis: A Pilot Study

Isha Khanduri ^1^, Dipen M. Maru ^2^ and Edwin R.Parra ^1,^*

*Correspondence: Edwin Roger Parra, MD, PhD, Associate Professor, Department of Translational Molecular Pathology, The University of Texas MD Anderson Cancer Center, Houston, Texas; email: [erparra@mdanderson.org](mailto:erparra@mdanderson.org)

**Supplementary Table 1: Antibodies clones of markers used in the multiplex immunofluorescence panel**

| **Antibody** | **Clone** | **Dilution** | | **Company** | **OPAL Dye spectra (nm)** |
| --- | --- | --- | --- | --- | --- |
| CK | AE1/AE3 | 1:25 | Dako | | 540 |
| CD68 | PG-M1 | 1:25 | Abcam | | 520 |
| CD163 | 10D6 | 1:100 | Leica Biosystems | | 690 |
| CD86 | E2G8P | 1:100 | Cell Signaling Technology | | 620 |
| CD206 | PA5-83759 | 1:100 | Thermo Fisher | | 570 |
| PD-L1 | E1L3N | 1:100 | Cell Signaling Technology | | 480 |
| ARG-1 | D4E3M^M^ | 1:200 | Cell Signaling Technology | | 650 |
| MRP8-14 | 27E10 | 1:50 | Abcam | | 780D |

**Supplementary Table 2: Phenotypes of the macrophage subtypes in the multiplex immunofluorescence panel**

| **Cell type** | **Phenotype** |
| --- | --- |
| Malignant cells | CK+ |
| Total macrophages | CD68+ |
| M1 | CD68+MRP8-14+CD163 **^neg^** CD206 **^neg^** Arg1 **^neg^** |
| M1 | CD68+CD86+CD163 **^neg^** CD206 **^neg^** Arg1 **^neg^** |
| M1 | CD68+CD86+MRP8-14+CD163**^neg^** CD206 **^neg^** Arg1**^neg^** |
| M2 | CD68+CD163+MRP8-14 **^neg^** CD86 **^neg^** |
| M2 | CD68+CD206+MRP8-14 **^neg^** CD86 **^neg^** |
| M2 | CD68+CD163+CD206+MRP8-14 **^neg^** CD86 **^neg^** |

All the macrophage phenotypes were co-localized with PD-L1 to quantitate PD-L1 expression on all the above macrophage phenotypes.
